# Structure, Function and Dynamics in Adenovirus Maturation

**DOI:** 10.3390/v6114536

**Published:** 2014-11-21

**Authors:** Walter F. Mangel, Carmen San Martín

**Affiliations:** 1 Biological, Environmental and Climate Sciences Department, Brookhaven National Laboratory, Upton, NY 11973, USA; E-Mail: mangel@bnl.gov; 2 Department of Macromolecular Structure and NanoBiomedicine Initiative, Centro Nacional de Biotecnología (CNB-CSIC), Darwin 3, Madrid 28049, Spain

**Keywords:** adenovirus, protease, DNA sliding, virus stability, uncoating, infectivity

## Abstract

Here we review the current knowledge on maturation of adenovirus, a non-enveloped icosahedral eukaryotic virus. The adenovirus dsDNA genome fills the capsid in complex with a large amount of histone-like viral proteins, forming the core. Maturation involves proteolytic cleavage of several capsid and core precursor proteins by the viral protease (AVP). AVP uses a peptide cleaved from one of its targets as a “molecular sled” to slide on the viral genome and reach its substrates, in a remarkable example of one-dimensional chemistry. Immature adenovirus containing the precursor proteins lacks infectivity because of its inability to uncoat. The immature core is more compact and stable than the mature one, due to the condensing action of unprocessed core polypeptides; shell precursors underpin the vertex region and the connections between capsid and core. Maturation makes the virion metastable, priming it for stepwise uncoating by facilitating vertex release and loosening the condensed genome and its attachment to the icosahedral shell. The packaging scaffold protein L1 52/55k is also a substrate for AVP. Proteolytic processing of L1 52/55k disrupts its interactions with other virion components, providing a mechanism for its removal during maturation. Finally, possible roles for maturation of the terminal protein are discussed.

## 1. Adenovirus

Adenoviruses (AdVs) [1] are among the most complex non-enveloped, icosahedral viruses. They have been found in most types of vertebrates, from fish to humans [2]. For historical reasons, and also because of their dual character as pathogens and therapeutic tools [3,4,5], the best characterized AdVs are those infecting humans, in particular the prototypes of human AdV (HAdV) species C, HAdV-C5 and HAdV-C2. Although different AdV species share many common traits, it must be understood that most of the information reviewed here has been derived from studies on these two prototypes, and details may vary (or are still unknown) for other human or non-human AdVs.

The AdV capsid is an icosahedron of ~950 Å maximum diameter and triangulation number *pseudo* T = 25 (see [6] for a description of the concepts “triangulation number” and “quasi-equivalent interactions”. For a detailed explanation on the adenovirus triangulation number, see [7]). Each capsid facet has 12 trimers of the major coat protein, hexon. A pentamer of penton base protein sits at each vertex, in complex with a trimer of the projecting fiber (300 Å-long in HAdV-C5). In addition, correct assembly requires four different minor coat proteins: IIIa, VI and VIII on the inner capsid surface, and IX on the outer one (reviewed in [7]). Minor coat proteins, together with flexible termini in hexon and penton base, modulate the quasi-equivalent icosahedral interactions and make up an intricate network that only recently could be visualized in detail via both X-ray crystallography and cryo-electron microscopy (cryo-EM) [8,9]. HAdV-C5 is the largest complex ever solved at high resolution (~3.5 Å) by either of the two techniques. Further witness to this complexity is the fact that even after being solved by two different techniques with close-to atomic resolution, the location of some of the minor coat proteins is still a subject of debate [7,10,11]. One issue is whether polypeptide IIIa is externally located. For the purpose of this review, we will follow the structural work indicating that it is internal [8,12,13,14], since this location is in better agreement with evidence indicating that IIIa interacts with the maturation protease (see below) and with the viral genome [15,16], and is released together with other internal vertex components in the early stages of virus entry [17].

The icosahedral shell encloses a non-icosahedral core composed of the linear, dsDNA genome (35 kbp in HAdV-C5), tightly packed in complex with a variety of DNA binding, viral proteins: core proteins V, VII and X (also called µ); the terminal protein (TP); and the maturation protease, AVP. Stoichiometric estimations indicate that from the 150 MDa total mass of the AdV particle, between 25 and 30 MDa are contributed by the core proteins [18,19]. There are no structural data on the core proteins (except for AVP, see below), and little is known regarding their organization within the particle, although it seems that polypeptide VII creates nucleosome-like beaded units that help to condense the genome so it can fit within the reduced capsid space [20,21].

The AdV infectious cycle starts with attachment to cell surface receptors (CAR for HAdV-C5) by the fiber distal domain [22]. Then, an RGD sequence motif in penton base binds to α_V_ integrins, promoting their clustering and triggering a signaling cascade that results in virus internalization by endocytosis [23]. Next, the viral particle travels from the cell membrane to the nuclear pore, while undergoing a stepwise uncoating process. The sequential uncoating starts at the plasma membrane, where upon binding to its receptor some fibers are released [24], and the penton base undergoes a conformational change that might result in weakening its interactions with the rest of the capsid [25]. Already at the membrane, and later on in the early endosome, vertex proteins are released, together with part of core protein V and protein VI [17,26,27,28,29]. Release of polypeptide VI is crucial, as this protein interacts with the endosomal membrane to promote its disruption and subsequent release of the AdV particle into the cytosol [27,30]. Although mild acidification in the early endosome may play a role in this second stage of uncoating, recent studies indicate that pH decrease is not required for entry of HAdV-C5/2 [31]. The partially disrupted virion associates with dynein motors via the hexon [32] and travels along the microtubular network until reaching the nuclear pore, where final dismantling occurs, and the viral DNA and core proteins enter the nucleus [33,34,35,36,37]. In the nucleus, the viral genome is transcribed into early mRNA, replicated, and finally late mRNA is synthesized. Newly synthesized capsid and core proteins are imported from the cytosol to the nucleus to assemble into new viral particles. The new genomes are packaged by an as yet unclear process, requiring the coordinated action of viral proteins IIIa, L1 52/55k, L4 33k, L4 22k and IVa2 between themselves and with the viral DNA packaging sequence [15,38,39,40,41,42,43,44,45]. One of these proteins, L1 52/55k, is present in empty capsids but must be released upon genome entry, as it is absent from the final virion. For this reason, it is considered a putative assembly scaffold [46]. Genome packaging produces the so-called young virions, which must be further processed by proteolytic maturation to yield the final, infectious AdV particle [47,48].

In AdV, correct uncoating is tightly linked to maturation. Young, immature virions are defective in uncoating. They cannot release fibers at the cell membrane, or polypeptide VI in the early endosome; consequently, they become trapped in the endocytic pathway, and are finally destroyed in lysosomes, thereby aborting infection [49,50]. Recent studies have provided new insights on the sophisticated mechanism of AdV proteolytic maturation and how this process modulates the stability of the viral particle, as well as the release of the putative scaffold, to confer the virion its full infectious character.

## 2. Players in Adenovirus Maturation: The Protease and Its Substrates

**Figure 1:**
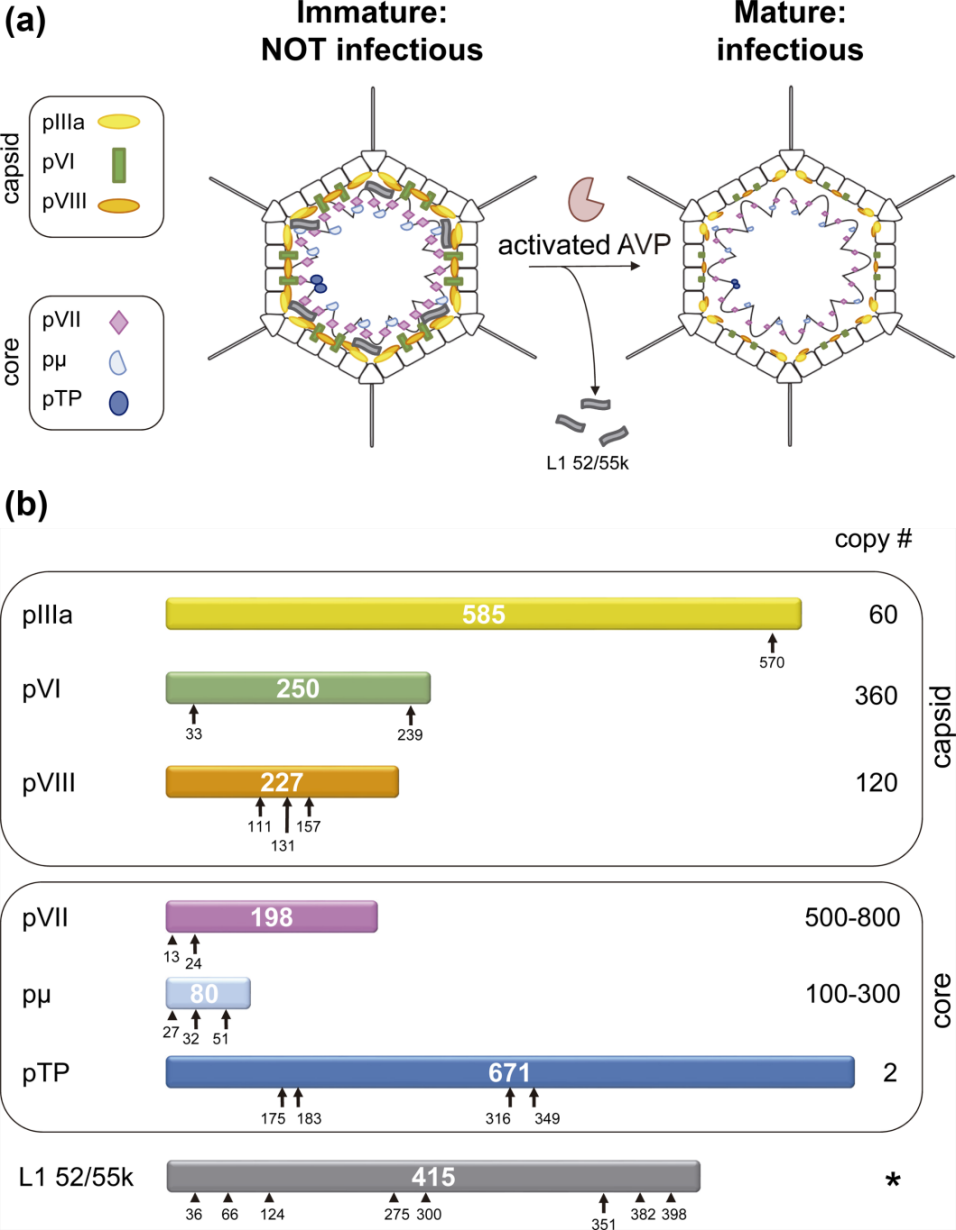
Substrates of the AdV maturation protease, AVP. (**a**) Schematics showing the location of substrates in the viral particle. The internal location of L1 52/55k is inferred from its interactions with core elements [59]; (**b**) Each HAdV-C2 precursor protein is represented as a bar with the polypeptide length in amino acids indicated in the center. Consensus cleavage sites are denoted by arrows, reported non-consensus sites by arrowheads [53,60]. The prefix “p” denotes the unprocessed precursors. Copy numbers are derived from stoichiometric analyses [18,19]. A star (*) in place of the copy number for L1 52/55k indicates that its copy number varies depending on the assembly stage: 100 copies in empty particles, 50 in fully packaged, immature *ts1* particles, and 0 in mature virions [46,60]. Panel (**b**) modified from [7].

AdV shell proteins IIIa, VI and VIII, as well as core proteins VII, µ and TP are synthesized as precursors, and processed by the adenovirus protease (AVP) during assembly [51,52,53,54]. The gene for AVP, which codes for a 23 kDa protein in HAdV-C5, is part of the L3 transcription unit and belongs to a conserved core of assembly-related genes present in all AdVs sequenced so far [55,56]. The locations of the protease substrates in the viral particle, as well as the cleavage sites and copy numbers for each of them are shown in Figure 1. Estimates on the copy number of AVP ranged between 10 and 50 [57,58]. A recent quantitative proteomics study gave an even lower number, with only seven AVP molecules per viral particle [19]. More than 2000 cleavages have to occur in each virion, giving a range of ~40 to ~300 cleavages per AVP copy. Furthermore, since all substrates are located internally and interact with the viral DNA, these cleavages have to take place in the highly crowded environment of the viral core.

An HAdV-C2 thermosensitive mutant, *ts1*, has served as an invaluable experimental system to elucidate the AdV maturation mechanism and its effect on infectivity. Weber and coworkers isolated the *ts1* mutant which, when grown at the nonpermissive temperature, contains precursor proteins instead of mature components [51]. The mutation was mapped to the gene encoding the 23 kDa L3 protein [51,61] which was later cloned and expressed in *E. coli* [57,62], and the resultant 204-amino acid protein purified [52,62,63,64]. The *ts1* mutation consists in the substitution of Proline 137 by Leucine in the AVP gene [65], and this mutation is both necessary and sufficient to generate the *ts1* phenotype [66]. The precise molecular effects of this mutation are not yet understood, but it is known that, when the virus is propagated at the non-permissive temperature (39 °C), the incorporation of AVP to the viral particles is minimal [51,61,65,66]. As a result, *ts1* particles are stalled at the young virion stage in morphogenesis: they accomplish genome packaging but do not undergo maturation; they contain the precursor versions of all AVP targets (L1 52/55k, pIIIa, pVI, pVIII, pVII, pµ, and pTP), and are not infectious.

**Figure 2:**
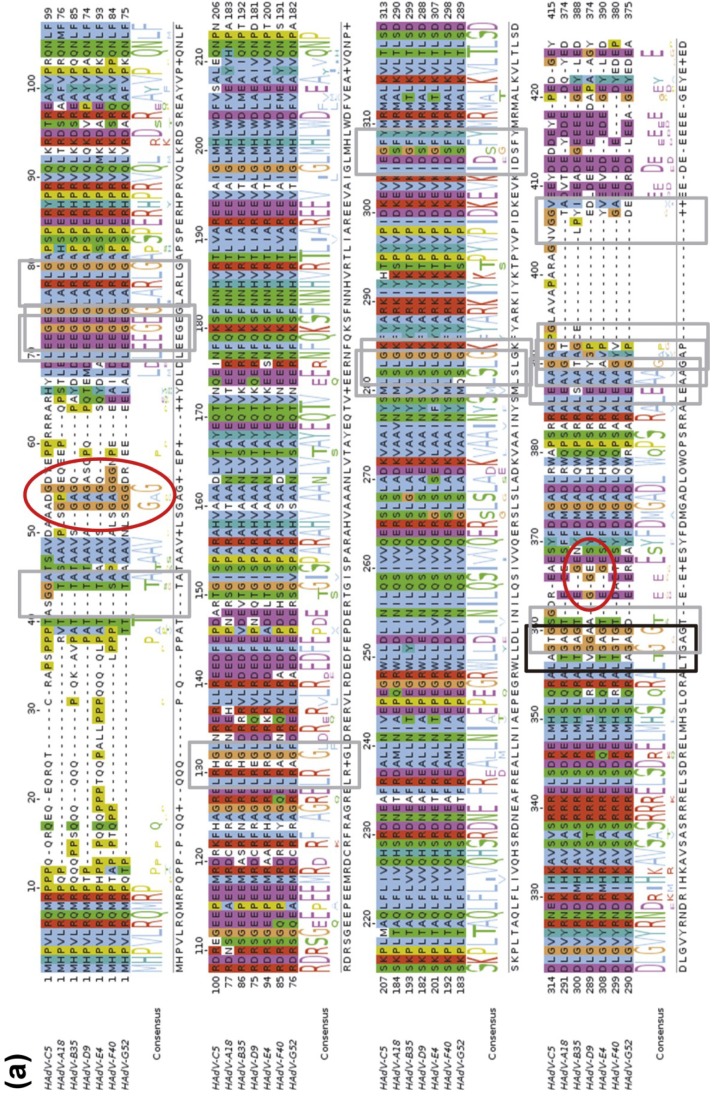

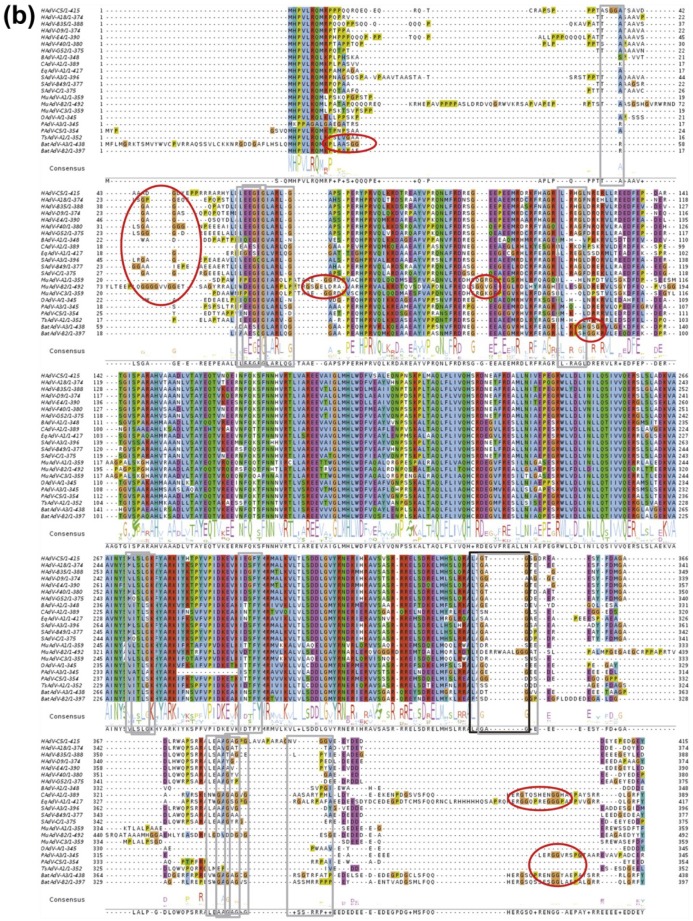
Conservation of AVP cleavage sites in the L1 52/55k protein. (**a**) Conservation across human AdV species; (**b**) Conservation across mastadenovirus species. Sequences were downloaded from GenBank and aligned with TCoffee [70]. The figure was created using JalView [71]. HAdV: human adenovirus; BAdV: bovine adenovirus; CAdV: canine adenovirus; EqAdV: equine adenovirus; SAdV: simian adenovirus; MuAdV: murine adenovirus; OAdV: ovine adenovirus; PAdV: porcine adenovirus; TsAdV: tree shrew adenovirus; BatAdV: bat adenovirus. Black frames indicate the AVP consensus cleavage site in HAdV-C2 L1 52/55k; gray frames indicate non-consensus sites described in [60]; red ovals indicate Gly stretches that could be possible additional cleavage sites.

AVP cleaves specifically at sequence motifs (M/I/L)XGG↓X or (M/I/L)XGX↓G [67,68]. However, these specificity requirements can be relaxed, as cleavages where the P_4_ residue is Gln or Asn, instead of Met, Leu or Ile were found by mass spectrometry analyses of HAdV-C5 [53]. In HAdV-E4, cleavage of pTP was observed at a site with Gln at P_4_ [69]. The packaging scaffold, L1 52/55k protein, had been predicted to undergo cleavage by AVP, based on the presence of a LAGT↓G motif close to its *C*-terminal end. Recent studies confirmed that indeed L1 52/55k is a substrate for AVP [60]. Immature *ts1* particles were shown to contain ~50 copies of full length L1 52/55k, indicating that although this protein is absent from mature virions, its presence is not incompatible with genome packaging. When these particles were treated with recombinant AVP, after mild disruption to gain access to the internal substrates, multiple fragments of L1 52/55k were generated, apart from the one expected from its consensus cleavage motif. Bioinformatics and mass spectrometry analyses indicated that AVP is able to cleave L1 52/55k at sites with various departures from the consensus cleavage sequences (Figure 1b). A comparison of the protein products of gene 52K indicates that the cleavage sites are highly conserved in human AdVs, and conservation is substantial throughout the Mastadenovirus genus (Figure 2). When some of the sites described for HAdV-C5/2 are missing, very frequently a series of Gly residues are observed in a nearby position in the sequence; these represent potential cleavage sites for the viral protease.

## 3. Unveiling the Enzymatic Mechanism of AVP

AVP has presented numerous conundrums on how its enzyme activity is regulated and how the active enzyme cleaves its substrates. What prevents the protease from being active after its synthesis but before completion of virion assembly? How is it activated? How can a few molecules of AVP cleave more than 2000 times within the tightly packed interior of a nascent virus particle, under conditions in which almost no three-dimensional diffusion can occur? Resolution of these conundrums revealed a new paradigm for virion maturation and a new type of biochemistry: one-dimensional biochemistry.

### 3.1. Discovery of the AVP Cofactors

A specific, sensitive, and quantitative assay for AVP using Rhodamine 110 as the reporting group [72,73] facilitated characterization of many of the biochemical properties of the enzyme. Rhodamine 110 is detectable at extremely low concentrations, because it has a molar absorbance coefficient greater than 70,000 M^−1^cm^−1^ and a quantum yield (the fraction of absorbed light that is emitted as fluorescence) greater than 90%. The assay is based on the observation that AVP will cleave small peptides that contain an AVP consensus cleavage sequence [68,74]. A fluorogenic substrate containing an AVP consensus cleavage sequence, (Cbz-Met-Arg-Gly-Gly-NH)_2_-Rhodamine, was synthesized and assays developed to characterize proteinase activity in disrupted wild-type virus [75,76]. Bis-substitution of Rhodamine 100 puts the Rhodamine moiety in the nonfluorescent, lactone state, because the conjugation in the Rhodamine moiety is interrupted. In the lactone state, the Rhodamine moiety does not absorb light and hence does not fluoresce. Cleavage of one of the two AVP consensus cleavage sequences generates mono-substituted Rhodamine; here, the Rhodamine moiety is in the highly fluorescent quinone state because the conjugation in the Rhodamine moiety is restored. Predictably, there was enzyme activity in disrupted wild-type virus and no activity in *ts1* disrupted virus. Surprisingly, no substrate hydrolysis was observed with purified recombinant AVP expressed in *E. coli*.

Eventually, it was shown that AVP needed cofactors for complete enzyme activity. When assayed with a Rhodamine-based substrate, neither recombinant AVP alone nor disrupted *ts1* virus alone exhibited enzyme activity. However, when mixed together, significant enzyme activity was observed [52,75]. Therefore, there are cofactors in the virus particle required by AVP for activity. Kemp and colleagues reached a similar conclusion after observing that although purified AVP cleaves the precursor to AdV protein VII, pVII, in the presence of *ts1* cell extracts, no cleavage of the peptide substrate SGGAFSW is detected with AVP alone [56]. One cofactor is the viral DNA, which stimulates AVP activity *in vitro* [75]. If disrupted wild-type virus is treated with DNase and then assayed with the synthetic substrate, proteinase activity is lost but, upon inactivation of the DNase, enzyme activity can be restored upon the addition of HAdV-C2 DNA [52]. A second cofactor is a plasmin-sensitive virion protein which turned out to be the 11-amino acid peptide, pVIc (GVQSLKRRRCF), from the *C*-terminus of the precursor to virion protein VI, pVI [56,75].

To investigate whether there was a nucleotide sequence specificity in the role of DNA as a cofactor, various nucleic acid and amino acid polymers were substituted for HAdV-C2 DNA in a series of cofactor assays [75]. Not only does T7 DNA substitute for HAdV-C2 DNA, but also single-stranded DNAs, circular single- and double-stranded DNAs, poly A, and even polymers of glutamic acid [77]. Neither polylysine nor the corresponding monomers of anionic polymers, such as AMP or glutamic acid, substitute for HAdV-C2 DNA [75]. Thus, there is no sequence specificity; rather, it appears as if the requirement is for a polymer of high negative-charge density, e.g., the viral DNA in the virus particle.

The cofactors affect the macroscopic kinetic constants for the interaction of AVP with the Rhodamine-based fluorogenic substrates [52]. In the absence of any cofactor, the *K_m_* is 94.8 μM and the *k_cat_* is 0.002 s^−1^. In the presence of AdV DNA, the *K_m_* decreases 10-fold and the *k_cat_* increases 11-fold. In the presence of pVIc, the *K_m_* decreases 10-fold and the *k_cat_* increases 118-fold. With both cofactors present, the *k_cat_*/*K_m_* ratio increases synergistically, 34,000-fold compared to that with AVP alone. Binding to DNA is coincident with stimulation of proteinase activity by DNA [78]. Other proteinases bind to DNA [79], but only the enzymatic activity of AVP is stimulated by being bound to DNA [52,75,78].

Interestingly, AVP has been shown to use a non-viral cofactor *in vivo*. Throughout an AdV infection, the actin, cytokeratin, tubulin, and vimentin networks that make up the cell cytoskeleton undergo dramatic changes [80]. Late in AdV infection, cytokeratin 18 is cleaved at two contiguous AVP consensus cleavage sequences, leading to the destruction of the cytokeratin network [81]. An AVP-GFP fusion transfected into HeLa cells was initially found in the cytoplasm where it colocalized with cytokeratin 18; later on in the experiment, the cytokeratin network was destroyed [82]. Thus, AVP can be active in the cytoplasm in the absence of other viral components. However, there must be a cytoplasmic cofactor ensuring activation of AVP, as incubation with AVP of cytokeratin-18 partially purified from the cytoplasm of HeLa cells resulted in no cleavage, while under the same conditions but in the presence of pVIc, cleavage of cytokeratin 18 was observed.

Actin was considered a potential cytoplasmic cofactor for AVP, because its *C*-terminal amino acid sequence (SGPSIVHRKCF) is highly homologous to the amino acid sequence of pVIc (GVQSLKRRRCF). Of the last eight amino acid residues of actin, four are identical and three are homologous to the last eight amino acid residues in pVIc. Furthermore, in the crystal structure of an actin-profilin complex [83], the *C*-terminus of actin is on the surface and could, therefore, be accessible to interact with AVP. Indeed, actin interacts directly with AVP [82]. When increasing concentrations of monomeric (G-)actin are incubated with AVP, the rate of substrate hydrolysis increases in proportion to the actin concentration until a plateau is reached, indicating that actin is indeed a cofactor for AVP. AVP binds to the *C*-terminus of actin, because the fluorescence from actin labeled with PRODAN at Cys374 is quenched upon incubation with AVP. The *K_d_* for the binding of AVP to actin is very tight, 4 nM. The role of actin as a cofactor in the cleavage of cytokeratin 18 was confirmed by the observation that when AVP and actin were incubated with a cytokeratin-18-enriched HeLa cell fraction, cleavage was detected. In an identical assay but without actin, no cleavage of cytokeratin 18 occurred. Inspection of the β-actin sequence revealed two AVP consensus cleavage sequences, one at the *N*-terminus and one at the *C*-terminus, raising the possibility that actin is not only a cofactor for AVP, but also a substrate. This possibility was verified by experiments in which actin and AVP were incubated together, and cleavage at the termini of actin was observed. In virus-infected cells, cleavage of cytoskeletal proteins weakens the mechanical structure of the cell. This weakening may promote cell lysis which is required for release of nascent virions [81].

### 3.2. Structure of AVP

**Figure 3:**
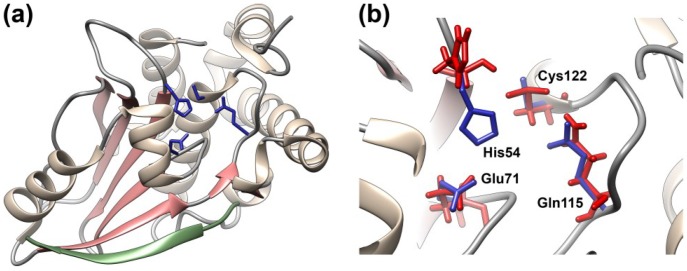
Crystal structure of the AVP-pVIc complex and locations of the four amino acid residues involved in catalysis in AVP and in AVP-pVIc. (**a**) Secondary structure of the AVP-pVIc complex with the four amino acid residues involved in catalysis in blue and the pVIc peptide in green; (**b**) The four amino acids involved in catalysis in AVP-pVIc (blue) and in AVP (red) are juxtaposed. Only His54 is in a different position in the two structures. Figure created with UCSF Chimera (http://www.cgl.ucsf.edu/chimera/) [90].

When AVP was first described, it was difficult to place it in any particular family of proteases. The sequence of the AVP gene [84,85] was not related to any gene sequences in the databases at the time. Inhibitor profiles of enzyme activity gave ambiguous results. The answer came from the crystal structure of the enzyme in a covalent complex with its cofactor pVIc [86,87,88]. The AVP-pVIc structure is ovoid, appearing to consist of two domains (Figure 3a). One domain contains a five-stranded β-sheet; the other domain contains mostly α-helices. pVIc forms a “strap” that helps position the two domains. Comparing the structure of AVP-pVIc with the structures of all unique protein molecules in the Brookhaven Protein Data Bank revealed no equivalent structure, suggesting that AVP represented a new family of protein molecules. However, a helix and several β-strands within the central region of AVP appeared to be in similar positions in papain [89]. When the common secondary structures were aligned, and the amino acids of the active-site region of papain and those in the same position in the AVP-pVIc complex were compared, it was clear what type of proteinase AVP was, as well as the location of its active site.

AVP was the first member of a new class of cysteine proteinases. The four amino acids involved in catalysis by papain have identical counterparts in the same relative positions in the AVP-pVIc complex [87] (Figure 3b). Cys122 of AVP is in an identical position to the nucleophilic Cys25 of papain. Two other residues of AVP (His54 and Glu71) are in identical positions to those of His159 and Asn175 in papain which have been shown to be involved in catalysis [91]. Even Gln19 of papain, presumed to participate in the formation of the oxyanion hole [92], aligns with Gln115 of AVP. The putative active site is on the surface of AVP lying within a ~25-Å long bent groove that is ~8-Å wide. Cys122 and His54, the general base, lie in the middle of that groove. Even with these juxtapositions, because the order along the polypeptide chain of the amino acids involved in catalysis in AVP and papain is different, AVP is the first member of a new class of cysteine proteases. This remarkable juxtaposition of catalytic elements in a groove that can accommodate substrate, strongly suggests that AVP employs the same catalytic mechanism as papain [93].

Surprisingly, pVIc, which exerts powerful control on the rate of catalysis, was found to bind quite far from the active-site residues involved in catalysis; the pVIc cysteine residue, which forms a disulfide bond with Cys104 of the AVP chain, is 32 Å away from the active-site nucleophile Cys22. The residue of pVIc closest to the active site is Val2', of which the side chain is 14.5 Å from Cys122. There is a reason for this long distance between the active site and the pVIc binding site, which is related to how AVP-pVIc complexes encounter their substrates and cleave them (see below).

**Figure 4:**
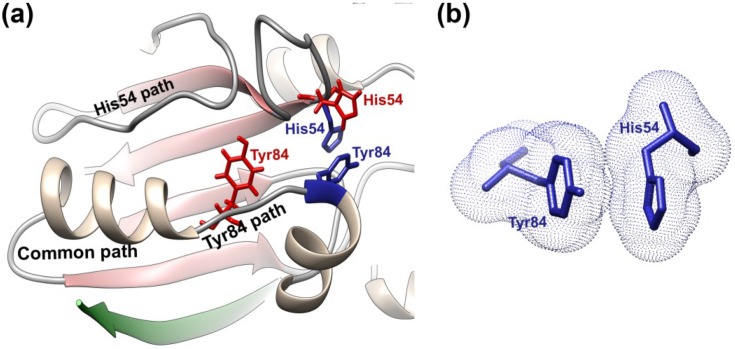
AVP activation pathway and cation-π interaction. (**a**) Upon binding of pVIc (green) to AVP, a series of contiguous conformational changes occur along a common path that bifurcates into an upper and lower path. At the end of the upper path, His54 drops down from its position in AVP (red) to a position in AVP-pVIc (blue) that is opposite Cys122. At the end of the lower path, Tyr84 (red) moves 11 Å to a position in AVP-pVIc (blue) where it can form a cation-π interaction with His54; (**b**) Electron clouds of Tyr84 and His54 in their cation-π interaction. Figure created with UCSF Chimera [90].

Later on, AVP was crystallized in the absence of any cofactor, and its structure solved to atomic resolution, 0.98 Å [94]. Both the crystal structure of AVP and the AVP-pVIc complex have an α plus β fold; the major structural differences between them lie in the β-sheet domain. Now, the structure of the inactive form of the enzyme could be compared to that of the active form, the AVP-pVIc complex [78,87], hopefully revealing at the structural level why AVP is inactive and providing insights as to how binding of pVIc to AVP activates the enzyme. In AVP-pVIc, the general base His-54 Nδ1 is 3.9 Å away from the Cys-122 Sγ; this distance allows the proton on Cys122 to be abstracted, thereby rendering Cys122 nucleophilic. In AVP, however, His-54 Nδ1 is 7.0 Å away from Cys-122 Sγ, too far away to be able to abstract the proton from Cys-122 (Figure 3b). The new structure revealed a fifth amino acid involved in catalysis. In AVP-pVIc, Tyr84 forms a cation-π interaction with His54 (Figure 4b). A cation-π interaction is a noncovalent molecular interaction between the face of an electron-rich system, e.g., Try84, and an adjacent cation, e.g., His54; between a monopole (cation) and a quadrupole (π system). Bonding energies are significant, with solution-phase values of the same order of magnitude as hydrogen bonds and salt bridges. The cation-π interaction between Tyr84 and His54 should raise the pK_a_ of His54 and freeze the imidazole ring in the optimal place for forming an ion pair with Cys-122. In AVP, however, Tyr84 is more than 11 Å away from its position in AVP-pVIc. The differences in position of His54 and Tyr84 are two major reasons why AVP is inactive and AVP-pVIc is active.

pVIc appears to function as a strap holding together the domain containing Cys122, with the other domain containing His54 and Glu71, in a configuration optimal for catalysis [95,96]. There is extensive contact between AVP and pVIc: 34 hydrogen bonds, four ion pairs, and a disulfide bond between Cys104 of AVP and Cys10' of pVIc [88,97,98]. The *N*-terminus of pVIc (Gly1', Val2', and Gln3') binds in a pocket, the “NT-pocket,” which is an invagination within the helical domain of AVP. Binding displaces a well-ordered sodium atom in the NT-pocket. That this pocket is structurally conserved between AVP and AVP-pVIc implies that perhaps the first step in the interaction of pVIc with AVP is the binding of the *N*-terminus of pVIc in this pocket. The binding of the next three amino acids of pVIc (Ser4', Leu5', and Lys6') also does not alter the structure of AVP; only surface side chain movements are necessary to accommodate these residues that bind as an extended β-strand. It is at Arg-7' and beyond that the binding of pVIc begins to induce significant rearrangements in AVP. These changes are: formation of a disulfide bond between Cys10' of pVIc and Cys104' of AVP and the formation of a new pocket, the “CT-pocket.” In the induced CT-pocket, which is hydrophobic, the side chain of pVIc Phe11' is buried.

pVIc can form a homodimer via disulfide bond formation, and half of the homodimer can covalently bind to AVP via thiol-disulfide exchange [56,99]. Alternatively, monomeric pVIc can form a disulfide bond with AVP via oxidation [99]. Regardless of the mechanism by which AVP becomes covalently bound to pVIc, the kinetic constants for substrate hydrolysis are the same. The cysteine residue in pVIc is clearly involved in the binding of pVIc to AVP. For example, the *K_d_* for the binding of pVIc to AVP is 4.4 μM, but the *K_d_* for the binding of the mutant C10'A-pVIc to AVP is much greater than 440 μM, at least a 100-fold difference. Surprisingly, the presence of DNA suppressed the effect of the mutation; the *K_d_* for the binding of the mutant C10'A-pVIc to AVP is 6.94 μM in the presence of 12 mer ssDNA. Although the cysteine in pVIc is important in the binding of pVIc to AVP, formation of a disulfide bond between pVIc and AVP is not required for maximal stimulation of enzyme activity by pVIc. However, covalent attachment of pVIc to AVP is physiologically relevant, because in the virus particle AVP is linked to pVIc via a disulfide bond [99,100]. The major function of the disulfide bond may be to keep AVP irreversibly activated inside the virion. If the free concentration of pVIc is low relative to its *K_d_* for binding to AVP, then reversible binding of pVIc to AVP might not be able to generate enough active enzyme to cleave all the virion precursor proteins. One way to ensure sufficient activation is the formation of an irreversible bond, e.g., a disulfide bond, between pVIc and AVP.

### 3.3. Interactions of AVP with Its Cofactors

In protein-protein binding, the free energy of binding at the level of amino acid side chains is typically not distributed evenly across the interface, but is contributed disproportionately by certain amino acids known as hotspots [101]. This is true for AVP-pVIc, as a small subset of buried amino acids contributes the majority of binding affinity of pVIc to AVP. This was determined by measuring the change in free energy, ΔΔG_B,_ upon mutation of individual residues in pVIc to alanine [97,98]. The two hot spots in pVIc are Gly1' and Phe11'. The $\Delta \Delta {\text{G}}_{T}^{*}$ upon substitution of an alanine for Gly1' is 1.57 kcal/mol, and for substitution of an alanine for Phe11', the $\Delta \Delta {\text{G}}_{T}^{*}$ is 1.15 kcal/mol [97,98]. Gly1' and the side chain of Phe11' are both buried in a pocket in the crystal structure [87,88]. Both residues are largely sequestered from solvent in the complex, with only 20% of the surface area of Gly1' accessible and 9% of the surface area of the Phe11' side chain accessible [97,98]. Val2' is also sequestered from bulk solvent, with only 0.01% of its side chain surface area accessible. The solvent occlusion of the hot spots in pVIc is consistent with studies of protein-protein interfaces, showing that solvent exclusion is a necessary condition of tight binding [101]. That the first and last amino acids of pVIc are hotspots is consistent with the hypothesis that pVIc acts as a strap that brings the two domains of AVP into alignment optimal for efficient substrate hydrolysis [95,96].

In general, it seems as if the *N*-terminus of pVIc is involved in the binding of pVIc to AVP and the *C*-terminus of pVIc is involved in stimulation of AVP activity by pVIc. More specifically, Gly1' is the major determinant in the binding of pVIc to AVP, while Phe11' is the major determinant in stimulating enzyme activity. The *N*-terminus of pVIc binds in a preexistent pocket whereas the *C*-terminus of pVIc binds in an induced pocket. These most crucial amino acid residues in the binding of pVIc to AVP and in stimulating the activity of AVP are conserved or tolerate only homologous substitution. The strictly conserved amino acid residues in pVIc are Gly1' and Cys10' [97,98]. Gly1' is conserved because it is part of the AVP consensus cleavage sequence, IVGL↓G; cleavage of pVI at this sequence liberates pVIc. Gly1' is also conserved because no amino acid side chain can fit into the hairpin of AVP that is the binding site for Gly1' [87,88]. The two other hot spot amino acid residues, Val2' and Phe11', tolerate only hydrophobic substitutions.

The effects of alanine substitutions in pVIc on binding to AVP are reversed in the presence of DNA. For example, the *K_d_* of Gly1'Ala-pVIc for AVP is 56 μM. In the presence of DNA, the *K_d_* drops to 0.08 μM, the same *K_d_* (90 nM) as for the binding of wild-type pVIc to AVP in the presence of DNA. For the alanine mutants of pVIc that exhibit *K_d_* values for binding to AVP lower than that for wild-type pVIc, the presence of DNA raises the *K_d_* values to that of wild-type pVIc. For example, the *K_d_* for AVP with the Gln3'Ala-pVIc mutation is 0.04 μM, compared to 4.4 μM with wild-type pVIc. In the presence of DNA, the *K_d_* for the mutant peptide is 0.13 μM, compared to 0.09 μM for wild-type pVIc. For substrate hydrolysis, the presence of DNA has little effect on the *K_m_* values; however, it does affect *k_cat_* values, up to 10-fold. At the moment, reversal by DNA of the effects of alanine substitutions on the amino acids in pVIc is a bizarre observation. Its explanation at the structural level should be novel.

AVP, pVIc, and AVP-pVIc complexes bind to DNA with physiologically relevant *K_d_* values. AVP was shown to bind a 12-mer ssDNA with a *K_d_* of 109 nM, and a 12-mer dsDNA a *K_d_* of 63 nM [78]. pVIc, with four of its 11 amino acid residues being basic and with an isoelectric point of 11.81, predictably binds to DNA; the *K_d(apparent)_* is 0.7 μM for binding to a 12-mer dsDNA. This may be physiologically relevant in that pVI, a DNA-binding protein [102], may bind to DNA via pVIc which is at its *C*-terminus. AVP-pVIc complexes exhibit a *K_d_* of 5 μM for 12-mer dsDNA and 109 μM for 12-mer ssDNA [78]. Consistent with the observation that binding of AVP to DNA is not sequence specific are data on the stoichiometry of binding. Three AVP-pVIc molecules saturate the binding sites on one 18-mer dsDNA, and six AVP-pVIc molecules saturate the binding sites on one 36-mer dsDNA. This implies the footprint on DNA is about six base pairs. On HAdV-C2 DNA, there are, therefore, about 3027 binding sites for pVIc.

The non-sequence specific interaction between AVP-pVIc and DNA exhibits a substantial dependence on the monovalent sodium ion concentration [52]. This dependence reflects the electrostatic component of the binding reaction [103]. The electrostatic component of the binding reaction originates from the formation of ion pairs between positively charged groups on AVP-pVIc and negatively charged phosphate groups on DNA. After binding occurs, there is a concomitant release of counterions from the DNA and, possibly, from AVP-pVIc. An accurate estimate of the number of ion pairs involved in the interaction was obtained from an analysis of the equilibrium association constants for the binding of AVP-pVIc to 12-mer dsDNA as a function of the Na^+^ concentration [78]. Two ion pairs are involved in complex formation with AVP-pVIc and 12-mer dsDNA. For comparison, two ion pairs of the T4 gene *32* protein are involved in non-sequence specific binding to helical DNA [103]. There is also a substantial favorable nonelectrostatic component of the binding interaction of AVP-pVIc to DNA [78]. The nonelectrostatic free energy of binding $\Delta {\text{G}}_{0}^{0}$ is −4.6 kcal/mol. These experiments indicate that much of the binding free energy under physiological conditions results from nonspecific interactions between AVP-pVIc and base or sugar residues on the DNA. But, the dominant factor driving the nonspecific interaction between AVP-pVIc and DNA is the entropic contribution from the release of counterions.

### 3.4. AVP Activation Pathways

A model has been proposed for the activation of AVP upon the binding of pVIc that is consistent with the structural differences between AVP and AVP-pVIc complexes [94].The structural changes that occur upon the binding of pVIc to AVP are localized for the most part to the β-strand domain and appear to involve a path over 62 amino acids long. This implies there may be an “activation” pathway along which contiguous conformation changes occur, analogous to falling dominos. The model (Figure 4) proposes that upon the binding of pVIc to AVP, a series of structural transitions occur in AVP, beginning with the induction of the CT-pocket. There is a common pathway that then bifurcates into pathways that lead to the repositioning of His54 and of Tyr84, the two amino acids in AVP that must be reoriented for the AVP-pVIc complex to become active.

The activation pathway is triggered when the three *N*-terminal amino acids of pVIc (Gly-Val-Gln) bind in a preformed, hydrophobic pocket, the NT-pocket, on AVP. Beginning with Leu5', the remaining amino acids of pVIc lay down upon AVP as an extended β-strand. Cys10’ of pVIc forms a disulfide bond with Cys104 of AVP. The *C*-terminal amino acid, Phe11', binds in an induced, hydrophobic pocket. The differences in the structure of AVP and the AVP-pVIc complex indicate that pVIc binding causes an extension in the S5 β-strand of the β-sheet by three amino acids: Cys104, Ile105, and Ser110. The CT pocket formation is coincident with the formation of the tight turn involving residues 100–103. The *C*-terminus of the long helix is extended from Ser95 to Ser99. Then, this portion of the helix rotates approximately 20 degrees from the long helix axis and changes its pitch by a similar amount. This is the common activation pathway.

The extension of the lower end of the long helix by a full turn and its movement alter the positions of its side chains and their interactions with the residues in the coil connecting strands S1 and S2. This results in repositioning of the backbone between amino acid residues 26–33, as indicated by the extensive phi and psi angle differences observed between the two structures. With these changes in the AVP structure, different portions of the coil interact with the repositioned helix, and other residues are now in positions to interact with the region of the undefined loop between residues 47–52 in AVP. This change allows hydrogen bonding to occur between residues 26–28 and residues within the undefined loop such that it now becomes much less flexible. A further consequence of this rearrangement is that the backbone moves, allowing the phi/psi rotation of the His54 backbone, which would enable its side chain to drop down to a position where it forms a hydrogen bond to Glu71 and is in a more optimal position for interacting with the active site nucleophile Cys122.

Triggering of the common activation pathway also initiates changes in the Tyr84 branch of the activation pathway. At Tyr88, the long helix breaks, extending into a coil through Tyr84. The extension of this portion of the long helix into a coil, along with the anchoring of its lower end due to the disulfide bond formation with pVIc and the “tethering” of the *N*-terminal portion of the helix to the central strand of the beta sheet, makes the *N*-terminal portion of the long helix rotate roughly 105 degrees generating the helix-coil-helix motif of AVP-pVIc. This movement also completes the formation of the active site groove across the domain interface. These events allow Tyr84 to move almost 11 Å so that it can now form a cation-π interaction with His54.

### 3.5. AVP Activation in Its Biological Context

AVP is synthesized as an inactive enzyme, which raises the question how pVIc is cleaved from pVI inside immature particles to activate AVP, *i.e.*, to form AVP-pVIc complexes. Restricting any model for the activation of AVP by pVI in such particles is the inability of AVP and pVI to undergo bimolecular interactions by diffusion in three-dimensional space. Both AVP and pVI are sequence-independent DNA binding proteins [75,78,97,102,104]. The high concentration of DNA inside the virion (>500 g/L) [105] forces both AVP and pVI onto the DNA. For AVP and pVI, the DNA-bound state predominates by at least 10-million-fold over the unbound state [78,106], meaning that most of the time, essentially none of these protein molecules is present in solution in the virion. This situation would diminish their three-dimensional diffusion constants by a similar factor. Additionally, the compacted DNA forms a tight mesh [106] that retards diffusion of the few molecules appearing in solution by 10-fold or more. From these points of view, inside the virion, the three-dimensional diffusion constants of these proteins are reduced by more than eight orders of magnitude relative to in a buffer solution with no DNA. The DNA genome cannot move to enable two DNA-bound proteins to interact. The friction on the capsid shell of the virion by densely-packed DNA immobilizes the DNA and the proteins bound to it. Given this situation inside the virion, it is not clear how a bimolecular interaction between AVP and pVI can occur that leads to cleavage of pVI and activation of the enzyme by released pVIc. Without this occurring, the virus particle cannot become infectious.

To elucidate in detail the mechanism of pVI cleavage and AVP activation by pVIc, the gene for pVI was cloned and expressed in *E. coli*, and the resultant protein purified and characterized [104]. pVI is a monomer at nanomolar concentrations, and binds very tightly to dsDNA independently of sequence. The *K_d(app)_* for binding of pVI to DNA is 46 nM in the presence of 1 mM MgCl_2_; in its absence binding to DNA was too tight to determine a *K_d(app)_.* Several lines of evidence indicate that pVI binds to DNA mostly through its pVIc moiety: First, AVP-pVIc complexes also bind tightly to DNA, with a *K_d(app)_* of 4.6 nM in the absence of magnesium [78]. Both proteins VI and AVP bind less tightly to DNA [78,104]. Their *K_d(app)_* values are almost 10-fold higher, 307 and 63 nM, respectively. Second, the number of base pairs covered while bound to DNA is similar for the complete pVI precursor (8 bp) and for AVP-pVIc complexes (6 bp). In contrast, the virion precursor protein pIIIa covers 33 bp [107]. Third, some thermodynamic parameters of pVI binding to DNA are similar to those of AVP-pVIc complexes binding to DNA [78,104]. The number of ion pairs formed in the binding to 12-mer dsDNA is three, whereas two ion pairs are involved in the interaction of AVP-pVIc complexes with DNA. The nonelectrostatic free energy of binding, $\Delta {\text{G}}_{0}^{0}$, is −4.5 kcal/mol, identical to that of AVP-pVIc complexes binding to DNA.

When AVP is mixed with pVI, no enzymatic activity is detected, even when both components are present at μM concentrations [108]. Incubation of AVP and pVI with 1 nM dsDNA results in 100% of pVI being cleaved, and all the AVP forming active AVP-pVIc complexes. Thus, activation of AVP to AVP-pVIc complexes by pVI requires the presence of DNA. Further analysis revealed that for AVP and pVI to interact, they both must be bound to the same DNA molecule.

The observation that both AVP and pVI must be on the same molecule of DNA for activation to occur in the absence of three-dimensional diffusion suggested that one of the molecules must slide into the other via one-dimensional diffusion along the DNA to promote the bimolecular interactions that lead to cutting out pVIc and its binding to AVP. Using total internal reflection fluorescence microscopy, AVP was observed binding randomly to phage lambda DNA, but not sliding [108]. In a similar assay, pVI was also observed binding randomly to DNA. However, once bound, pVI slid rapidly over tens of thousands of base pairs before dissociating from the DNA. The MSD (mean square displacement, the square of the distance traveled) for each molecule was linear with diffusion time, indicating transport dominated by Brownian motion. The mean one-dimensional diffusion constant was 1.45 ± 0.13 × 10^6^ bp^2^/s.

There is a specific sequence of events in the cleavage of pVI by AVP in the presence of DNA, both *in vitro* and *in vivo*. *In vitro*, pVI is initially cleaved at its *N*-terminus (releasing amino acids 1–33) and then at its *C*-terminus (releasing pVIc, amino acids 239–250). After the second cleavage, the released pVIc binds to the AVP that cut it out. An identical cleavage and activation sequence occurs in a quasi *in vivo* system with *ts1* virus grown at the nonpermissive temperature. Incubation of heat disrupted *ts1* particles with AVP results in the processing of pVI to protein VI. If disrupted virus is incubated with DNase and then AVP is added, no processing of pVI is observed. Most convincing, if disrupted virus is incubated with DNase, the DNase inactivated and DNA added back along with AVP, pVI undergoes processing to protein VI and AVP-pVIc complexes are formed [108].

### 3.6. AVP Function in Its Biological Context

Once the active AVP-pVIc complex has been formed, how can a few molecules of the active protease cleave at several thousand sites within the nascent particle? Like AVP, pVI, and the AVP-pVIc complex [75,78,97], the adenoviral precursor proteins pVI, pTP, pVII, pIIIa, pµ, and L1 52/55k are sequence-independent DNA-binding proteins [59,102,109,110,111]. The situation is not dissimilar to that faced by AVP and pVI in the formation of AVP-pVIc complexes, in that the active protease needs to reach its substrates when both AVP-pVIc complexes and their substrates are essentially irreversibly bound to a fixed matrix, the viral DNA.

**Figure 5:**
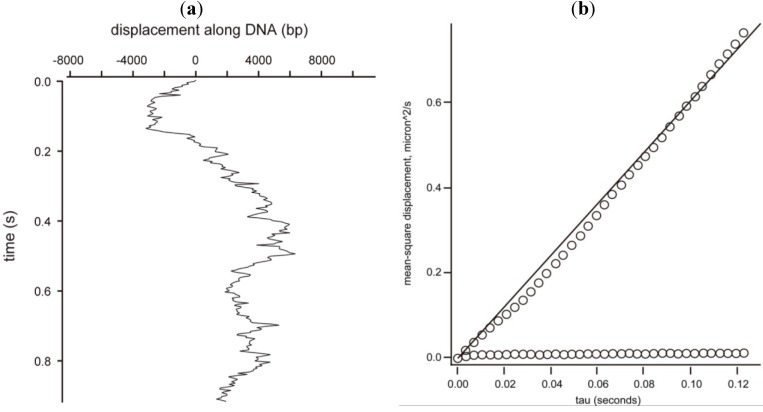
Sliding of the AVP-pVIc complex along DNA. (**a**) An AVP-pVIc complex sliding almost 16,000 bp in less than 1 s; (**b**) Mean square displacement (MSD) *versus* time of the data in (**a**). The MSD is the square of the distance traveled. The slope of the curve is the one-dimensional diffusion constant for this slide, 32 × 10^6^ bp^2^/s. The line parallel to the abscissa is the MSD from the *Y*-axis versus time.

Single-molecule fluorescence microscopy with flow-stretched DNA was used to determine whether AVP-pVIc complexes slide on DNA [16]. AVP-pVIc complexes bound randomly to DNA and were observed to diffuse rapidly over tens of thousands of base pairs (Figure 5). The mean one-dimensional diffusion constant was estimated as 21.0 ± 1.9 × 10^6^ bp^2^/s, and sliding exhibited Brownian motion. These observations implied that AVP-pVIc complexes may slide on DNA to encounter and process the virion precursor proteins also bound to DNA.

If sliding of AVP-pVIc complexes on DNA is required for the processing of virion precursor proteins, then one would predict that processing of virion precursor proteins would occur only in the presence of DNA [16]. The validity of this prediction was confirmed by experiments showing that *in vitro*, in the absence of DNA, no conversion of pVI to VI occurred upon incubation of AVP-pVIc complexes with pVI. In the presence of DNA, pVI was processed to VI in two steps; the same way it was processed by AVP in the presence of DNA [108]. *In vivo*, processing of the precursor proteins in heat-disrupted *ts1* virus by AVP-pVIc complexes was also found to be DNA dependent. When AVP-pVIc complexes were incubated with heat-disrupted *ts1* virus, all the virion precursor proteins were processed [16]. If, before adding AVP-pVIc complexes, the heat disrupted *ts1* particles were incubated with DNase, no processing of pVI or the other precursor proteins was observed. However, when heat disrupted *ts1* particles are incubated with DNase, the DNase inactivated by EDTA, and *ts1* viral DNA added back along with AVP-pVIc complexes, full processing of virion precursor proteins is observed.

Proteins can slide along DNA either by traveling straight down the double helix (simple one-dimensional translational diffusion) or by rotating along the DNA double helix while maintaining a specific orientation with respect to the DNA double helix. If a protein is constrained to move along the DNA helix (for instance, in order to maintain optimum contact between its DNA-binding patch and the surface of the DNA helix), the protein will be forced to rotate while translating. It was shown that AVP-pVIc complexes undergo rotation-coupled sliding along the DNA helix on a rugged free-energy landscape [112]. The average free-energy barrier for AVP-pVIc complexes sliding along the DNA is 1.1 ± 0.2 kBT. Such a small barrier facilitates rapid movement.

### 3.7. Using AVP as a Target for Anti-Adenovirus Drugs

Adenoviruses are the cause of numerous, significant diseases and antiviral agents against AVP would be valuable. In general, adenoviruses cause ocular, acute respiratory and gastrointestinal infections. Adenoviruses are responsible for approximately 5% of the acute respiratory diseases in children under the age of five [113] and for about 10% of the pneumonias of childhood [4,114]. They are also opportunistic pathogens in AIDS patients [115]. HAdV-B7 has been shown to be responsible for major outbreaks of acute respiratory disease among military recruits [116,117]. More recently, HAdV-D36 has been found to be associated with obesity [118], and a variant of HAdV-B14 infected more than 140 people on an island, 10 of whom died [119]. It is estimated that 20–40 million cases of epidemic keratoconjunctivitis arise each year, more than one million cases in Japan alone. While vaccines are highly effective in preventing viral infections, with other viruses, permanent, universal vaccines have been difficult to develop (e.g., HIV, flu, *etc.*). As there are more than 60 adenovirus serotypes, it may be difficult to get a universal, effective adenovirus vaccine. Antiviral agents can be useful for short-term protection, e.g., for self- limiting virus infections such as those that can be induced by adenovirus.

One antiviral agent has been identified based on the biology of AVP. Regulation of AVP activity requires that the enzyme is synthesized in an inactive form, because if it were active before virion assembly, it would cleave virion precursor proteins, thereby preventing virus morphogenesis. From this point of view, it can be predicted that pVIc could be turned into an antiviral agent if it were present before complete virion assembly. This hypothesis was tested by adding pVIc to virus-infected cells at various times after infection [97,98]. When pVIc was added between 4 and 16 h post infection, there was no reduction in the level of synthesis of infectious virus. However, when added at time zero along with virus, or beyond 20 h post infection, there was a large reduction in the synthesis of infectious virus, e.g., 99.8% at 28 h post infection.

A series of AVP inhibitors have also been developed based on the biology of the enzyme, and new methods for using inhibitors have been devised to prevent drug resistance [120,121,122]. *In silico* screening of a chemical database identified 2,4,5,7-tetranitro-9-fluorenone [123] as a potential inhibitor of AVP. This compound selectively and irreversibly inhibits AVP in a two-step reaction: reversible binding (*K_i_* = 3.09 μM) followed by irreversible inhibition (*k_i_* = 0.006 s^−1^). The reversible binding is due to molecular complementarity between the inhibitor and the active site of AVP which is the basis for the selectivity of the inhibitor. The irreversible inhibition is due to substitution of a nitro group of the inhibitor by the nearby Cys122 in the active site of AVP.

Comparison of the crystal structures of inactive AVP [94] and active AVP-pVIc complexes [86,87,88] reveals a number of differences which could be considered as targets for drug interactions. These sites, which cover more than 40% of the surface of AVP, include the active site [86,87,88,94], pVIc binding site, DNA binding region [96,101], and the activation pathway [94]. Using structure-based drug design, a lead compound was identified that was predicted to bind to both the active site and the conserved site at which the *N*-terminus of pVIc binds [124]. This compound is a competitive inhibitor with a *K_i_* of 0.43 μM. A derivative of this compound has an IC_50_ of 140 nM, and does not inhibit trypsin or papain at concentrations of enzyme up to 10 μM.

## 4. Effect of Adenovirus Maturation on the Viral Particle

Assembly and maturation in dsDNA viruses is most understood for the tailed bacteriophage and structurally related herpesviruses. In herpesvirus and in many bacteriophage, maturation is triggered by a viral protease and coupled with DNA packaging [125]. Large rigid-body movements of capsomers and dramatic capsid expansion allow the packaged genome to become part of a highly stable particle that will protect it from the environment until reaching a new host cell [126]. Bacteriophage and herpesvirus maturation are the obligate references when picturing dsDNA virus maturation. In AdV, however, studies on the structure and stability of the immature particle show a rather different picture.

### 4.1. Structural Changes Induced by Maturation of the Viral Particle

**Figure 6:**
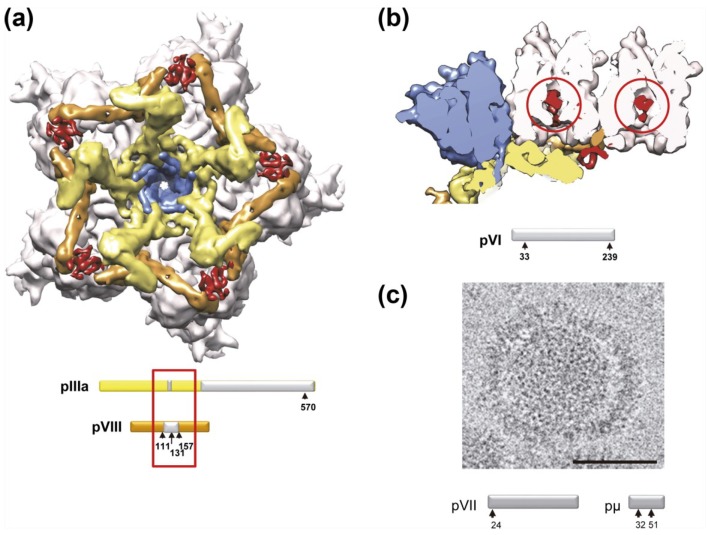
Structural differences between immature (*ts1*) and mature (wt) AdV virions. (**a**) View from inside the capsid looking at the 5-fold icosahedral symmetry axis, with the density for the molecular stitch derived from the *ts1*-wt difference map at 8.9 Å resolution in red [128]. The five peripentonal hexons are shown in pale pink; penton base in blue; polypeptide IIIa in yellow; and polypeptide VIII in tan. Surfaces in (**a**) and (**b**) were created from the HAdV-C5 high resolution cryo-EM structure (PDB ID 3IYN) [8] and represented with UCSF Chimera [90]. The bars represent the precursor polypeptides IIIa and VIII with the cleavage sites indicated (arrows). Polypeptide regions not traced in the cryo-EM HAdV-C5 high resolution structure are in gray. Untraced regions close to the molecular stitch are indicated with a red rectangle. Modified from [7]; (**b**) A section across the capsid showing the density attributed to the precursor of protein VI (red circles) inside the inner cavities of two hexon trimers [128]. Colors are as those shown in (**a**). Density for the molecular stitch is also seen in this view, wedged between polypeptide IIIa and VIII. The bar represents the precursor polypeptide VI with the cleavage sites indicated (arrows); (**c**) A disrupted particle found in a cryo-EM preparation of *ts1* virus, showing the capsid separating from the core, while the latter remains as a compact sphere. The bar in the micrograph represents 50 nm. The bars below represent the precursor polypeptides pVII and pμ with the cleavage sites indicated (arrows). Reproduced with permission from Reference [128]. Copyright 2009, Elsevier.

Two cryo-EM studies have analyzed the structural differences between mature (wild type, wt) and immature (*ts1* mutant at the non-permissive temperature) adenovirus particles, at resolutions in the subnanometer range [127,128]. It was evident that, unlike bacteriophage, AdV does not experience massive conformational rearrangements during maturation. However, three differences between the mature and immature particles were observed. First, on the inner capsid surface of *ts1,* extra densities located between the peripentonal hexons and those in the central plate of the facet were dubbed a “molecular stitch”, that is, a structure that would contribute to hold the vertex components in place during assembly, but is removed afterwards to facilitate vertex release for uncoating [128] (Figure 6a). The molecular stitch is in close proximity to polypeptide VIII, one of the substrates of AVP. It is directly adjacent to two regions where polypeptide chains no longer could be traced in a quasi-atomic resolution HAdV-C5 study by cryo-EM, either because of their absence or because of disorder [7,8]. These regions are: a short stretch of residues in polypeptide IIIa (residues 216–225), and the central fragment of polypeptide VIII produced by AVP cleaving at residues 112 and 157 (Figure 1). There are two independent copies of polypeptide VIII in the AdV asymmetric unit. One is located beneath the peripentonal hexons, in close contact with polypeptide IIIa, while the other is closer to the three-fold icosahedral axis. The molecular stitch was only observed close to the peripentonal copy of VIII. Therefore, it seems likely that this structure is formed by the contribution of the central peptide of uncleaved pVIII and IIIa.

The second difference observed consisted in additional density located inside all hexon cavities in the *ts1* structure (Figure 6b) [127,128]. Weak density has been observed at this location in the mature particle, and attributed to polypeptide VI [8,9,12]. In one of the cryo-EM studies on *ts1*, the extra density inside hexons was assigned to pVIc, based on size considerations [128]. More recent structural and molecular studies indicate that the part of polypeptide VI located within the hexon cavity may be the pVI *N*-terminal peptide, although in this case the density observed occupies a more external position in the hexon cavity than the extra density observed in *ts1* [10,129]. Nevertheless, stronger density in cryo-EM maps of *ts1* indicates that the interaction between polypeptide VI and hexon changes upon pVI cleavage by AVP, with a more uniform occupancy or ordering of the part of pVI within the hexon cavity prior to maturation. This interaction change relates to the lack of infectivity in immature AdV: a strong interaction with hexon established by the precursor would have to be loosened by maturation to facilitate release of protein VI from the capsid in the endosome.

The third difference observed between mature and immature AdV particles concerns core organization. Both cryo-EM studies indicated that the core undergoes a transition from a more ordered to a more disorganized structure during maturation [127,128]. Disrupted *ts1* virions released compact, spherical cores, hinting at an extra stabilization of the structure (Figure 6c) [128]. This observation suggests that precursor proteins pVII and pµ have a much stronger dsDNA condensing activity than their mature versions.

### 4.2. Stability Changes Induced by Maturation of the Viral Particle

Early studies on the *ts1* mutant had related its entry defect with increased stability of the virus particle [130]. The structure of the *ts1* mutant at 8.9 Å resolution revealed extra ordered densities and a tighter core condensation attributable to the presence of uncleaved precursors in the immature particle (Figure 6) [128]. These structural differences suggested changes in interactions between the components of the viral particle that would be related to the ability, or lack thereof, to uncoat. The actual effect of the different interactions on particle stability and disassembly was revealed by *in vitro* disruption studies [131].

Immature *ts1* particles are considerably more stable than the mature (wt) virions under a variety of stress sources (heat, acidification, denaturing agents), as indicated by differential scanning calorimetry, extrinsic fluorescence and electron microscopy analyses. Wild type capsid disassembly starts at mild conditions at which *ts1* remains unaltered: for example, at 40 °C in heat disruption experiments; or at pH 6 to 6.5, which interestingly, mimics the pH conditions in the early endosome. This is another difference with the maturation process in bacteriophage. Unlike the bacteriophage capsids, the mature AdV virion does not represent a global energy minimum in the assembly pathway. It is rather a metastable particle, primed for sequential disassembly through a series of irreversible events, and massive genome exposure under the appropriate conditions. These energetic differences are likely related to the different infection mechanisms used by bacteriophage and AdV. dsDNA bacteriophage translocate their genomes into the host cell leaving the protein shell behind [132], while AdV is engulfed by the cell and disassembles within to expose its genome to the cell nucleus machinery. In this sense, AdV is similar to other non-enveloped animal viruses, such as poliovirus, where interaction with the receptor is the trigger to start the cascade of structural changes leading to uncoating [133].

Maturation facilitates penton release under the appropriate conditions, e.g., mild acidification. Not all pentons are released at the same time; in particular, at pH 6 only one or two pentons per wt particle were found missing in cryo-electron tomography images [131]. Real time observations of the disassembly process induced by mechanical stress showed how pentons are sequentially released, and indicated that maturation reduced the energetic demands for penton ejection by a factor of 2 [134]. Penton loss is accompanied by loss of density in the peripheral core region, consistent with observations indicating release of some internal components in the early endosome, such as core polypeptide V and, more crucially, the membrane disrupting polypeptide VI [26,27]. A massive increase in DNA accessibility to intercalating dyes accompanies penton loss [131]. Interestingly, the viral genome also becomes accessible to click chemistry labels while the viral particle traffics within the cytosol in infected cells [36]. DNA accessibility in the first stages of disassembly results from penton defects and core reorganization, but not from core ejection, as most particles observed still retained electron dense contents, and only very rarely were short dsDNA stretches observed protruding from the virions [131,134]. These properties correlate with the need for the partially disassembled virion to keep protecting its genome while trafficking in the cytosol until arrival to the nuclear pore, while at the same time allowing access to cellular sensors triggering inflammatory responses [135]. DNA exposure to the environment occurred at higher levels of stress for the immature particle, and never reached the same cooperativity levels as in WT. At pH 5, when mature particles crack open and appear completely devoid of genome, the immature core remained forming a compact sphere attached to large capsid fragments [131,134]. These observations highlight the role of precursor proteins in bridging capsid to core, and in helping condense the viral genome so it can fit inside the capsid shell. On the other hand, maturation is required to prepare the viral particle for penton release, induce core relaxation and facilitate genome ejection at the nuclear pore.

### 4.3. Release of Packaging Scaffold Protein L1 52/55k

The unique mode of action of AVP and its dependence on dsDNA [16,75,108] imposes a tight link between the processes of genome packaging and maturation in AdV. One more link between these processes was recently discovered when the putative scaffold protein L1 52/55k was proved to be also a substrate for AVP [60]. The phosphoprotein L1 52/55k has been considered a possible scaffolding factor in AdV, because it is present in incomplete particles (devoid of the complete genome and considered packaging intermediates), but absent from the mature virion [46]. It would not be a classical scaffold protein however, since it does not seem to be required for capsid shell assembly [38]. Rather, it appears to be involved (as the other shell precursors) in establishing interactions that stably bridge shell and genome, acting as a packaging scaffold. Apart from having the ability to self-interact, L1 52/55k binds to the AdV packaging signal (through possibly an indirect interaction using another viral protein as intermediary) [42,44]; to the putative packaging ATPase IVa2 [136]; to the major core protein VII [59]; and to the shell protein IIIa [15]. The last two proteins are also processed by AVP during maturation. Interestingly, the multiple cleavages in L1 52/55k impair these interactions, facilitating its release from the nascent virion [60].

L1 52/55k is processed by AVP, but even after extensive digestion times (12 h) very large fragments (even the full length protein) are still present in the reaction, with sizes ranging between 47 and 17 kDa [60]. However, only traces of L1 52/55k are found in mature viruses [46,60]. How are the large fragments expelled from the immature virion? One possible mechanism is that L1 52/55k is further processed into smaller fragments by another, as yet unknown, maturation player. Alternatively, the release of these large fragments must occur at a time when there are still considerably large exit ways in the particle. As AdV does not undergo large conformational rearrangements upon maturation, it follows that L1 52/55k fragments must be released while the capsid shell is still assembling. For other AVP substrates however (pVI, pVII, pVIII, pre-µ), excised terminal peptides have been found to be present in mature virions by MS analyses [19,53,129], indicating that either they strongly bind to other partners in the particle, or their cleavage is taking place after the particle is sealed.

The presence of full length L1 52/55k protein in *ts1* had previously been considered negligible [46]. However, recent studies indicate that as much as 50 copies of L1 52/55k are present in the young virion even after full genome packaging [60]. This new observation suggests that unprocessed L1 52/55k may also be a factor in the immature AdV inability to uncoat, by preserving strong interactions between core and shell that preclude genome detachment at the nuclear pore.

## 5. Proteolytic Processing of Pre-Terminal Protein

AdV terminal protein is the only early protein processed by AVP (Figure 1). One copy of mature TP (37 kDa) is covalently bound to each 5' terminus of the dsDNA genome in the infectious virus particle [137], but TP is synthesized as a larger precursor (pTP, 76.5 kDa) [54,138]. Cleavage proceeds via an intermediate form (iTP, 56 kDa) that was observed in *ts1* virions grown at the permissive temperature (32 °C) [138]. iTP is short-lived in HAdV-C2 but can be readily detected *in vitro* in HAdV-E4, probably reflecting a lower efficiency of cleaving at the non-consensus site QRGF↓G [69]. pTP has four potential cleavage sites (Figure 1) in HAdV-C5, of which three have experimentally been observed [109]. The iTP intermediate is actually a mixture of the products obtained after cleavage at either residues 175–176 or 183–184, while cleavage at 349–350 produces the mature TP.

Although the crucial function of pTP in viral genome replication has been extensively studied (reviewed in [139]), the role of its proteolytic maturation in the viral cycle is less clear. It has been proposed that maturation of pTP is required to release the nascent virions from the nuclear matrix [140]. However, the fact that *ts1* immature particles are readily assembled, packaged and released is inconsistent with this hypothesis. Both pTP and TP, as well as iTP, can serve as primers to initiate viral genome replication [69,141,142]. Parental TP must be used in the first replication round after entry, while for subsequent rounds the newly synthesized pTP form will be available. Therefore, it cannot be ruled out that mature TP helps to make early replication more efficient, under conditions in which viral templates are still scarce. A third hypothesis is the requirement for pTP processing to help target it and its final product TP to different nuclear localizations. Immunofluorescence assays showed that the location of TP in the nucleus of HAdV-C2 infected cells is limited to a punctate pattern similar to that of early replication sites, while pTP was localized throughout the nucleus [69]. Further support for the role of TP in targeting of the entering genomes comes from recent work showing that a mutation changing Gly 315 to Val in HAdV-C5 pTP results in viral particles containing 10 times more iTP than WT. These particles could escape the endosome, but their genomes had difficulty reaching their destination in the nucleus and were degraded in the cytosol [143]. This study proved that processing of the fourth consensus site in pTP is relevant for AdV infectivity.

## 6. Concluding Remarks and Remaining Questions

### 6.1. Enzymology and Mode of Action of AVP

*Model on the role of AVP in virion maturation.* Detailed studies on the enzymology of AVP have resulted in the following model for its role in maturation: (a) AVP is synthesized in a catalytically inactive form [56,75]. If AVP were synthesized as an active enzyme, it could cleave virion precursor proteins before virion assembly, and this would abort the infection [97]; (b) Inside immature virions, AVP binds to the viral DNA [75,78]. Binding to DNA partially activates the enzyme [75,78]; (c) Although AVP bound to DNA does not slide on DNA, pVI does slide on DNA via one-dimensional diffusion [108]. pVI slides into AVP when both are bound to the same DNA molecule; (d) The partially activated AVP [52] cleaves pVI first at its *N*-terminus releasing a 33 amino acid peptide, and then at its *C*-terminus releasing pVIc [108]; (e) The released pVIc binds to and forms a disulfide bond with the AVP that cut it out; (f) AVP-pVIc complexes bind tightly to DNA, and the ternary complex, AVP-pVIc-DNA, is the most active form of the enzyme [52,78,97]; (g) Although AVP binds to but does not slide on DNA [108], the fully active protease, the AVP-pVIc complex bound to DNA, does slide along the DNA via one-dimensional diffusion [16]; (h) As the AVP-pVIc complexes slide along DNA, they process the precursor proteins also bound to the DNA. In summary, the pVIc peptide is a *molecular sled* used first as part of pVI to slide itself into DNA bound AVP; then the sled is cut out from pVI whereupon it binds to AVP to form the AVP-pVIc complex. The sled activates AVP and enables it to slide into the rest of its substrates to process them.

*Gaps in understanding how AVP functions at the molecular level.* To fully understand the molecular action of AVP, crystal structures of AVP, pVIc, AVP-pVIc, and pVI bound to DNA are required, as well as AVP-pVIc complexes in the presence of substrate plus and minus DNA. These structures will reveal at the structural level how DNA increases the activity of AVP and AVP-pVIc complexes; the structure of the sliding interface of AVP-pVIc complexes, pVIc, and pVI on DNA; the amino acids involved in sliding; the relative positions and orientation between the sliding interface, the active site and the substrate binding site; and the physical mechanism of sliding.

*Cleavage by sliding of AVP on “decorated” viral DNA.* A major unanswered question is: how can AVP and AVP-pVIc complexes slide on the viral DNA *in vivo*, given that the DNA is decorated with multiple copies of the seven different precursor proteins? That this sliding does occur has been shown by experiments with heat-disrupted *ts1* virions. Electron microscopy studies of mildly heat-disrupted *ts1* virions show that part of the viral DNA is extruded through a hole in the virion [128]. The width of the extruded filament implies that the DNA is decorated with precursor proteins. When AVP is added to mildly heat-disrupted *ts1* virions, the precursor proteins are processed [60]. It has also been shown that for AVP to be activated by pVI *in vitro*, not only is DNA required, but both AVP and pVI must be on the same DNA molecule. Thus, pVI and AVP-pVIc complexes slide on decorated DNA to interact with their substrates.

There are several different ways in which pVI and AVP-pVIc complexes could slide on decorated DNA to interact with their enzymes or substrates. The cleavage products of some of the precursor proteins may dissociate from the DNA after processing of the precursor form. For example, pVI has a *K_d_* of 46 nM for DNA, whereas its fully processed product, protein VI, has a *K_d_* of 397 nM [104]. It has been shown that DNA compaction driven by precursor core proteins is relaxed by maturation [128,131,134]. On the other hand, it is clear that protein VII remains bound to DNA after processing of pVII. Furthermore, protein V is also bound to the viral DNA but is not a substrate for AVP. It is possible that pVI and AVP-pVIc complexes can slide past other proteins bound to DNA, e.g., by sliding in a groove of the DNA, major or minor, in which the other proteins are not bound. A more likely possibility is that a combination of sliding along the DNA and hopping on and off the DNA is occurring. These hypotheses are currently being tested experimentally.

*New type of biochemistry: one-dimensional biochemistry*. The data on how AVP is activated and cleaves its substrates imply that a new type of biochemistry, one-dimensional biochemistry, is operative in AdV maturation. Some of the classic parameters characterizing bimolecular interactions are less meaningful in this new type of biochemistry. For example, pVI is not cleaved by highly active AVP-pVIc complexes in solution; AVP-pVIc complexes must slide along the DNA into pVI for a productive bimolecular interaction to occur [16]. In this case, equilibrium dissociation constants that characterize bimolecular interactions in three-dimensional space are less predictive of productive collisions than the individual equilibrium dissociation constants for the binding of the two components to DNA and the one-dimensional diffusion constants. AVP-pVIc complexes and their substrates bound to DNA are highly constrained, both in space and in orientation. That, plus the constraint that within the virus particle AVP-pVIc complexes move only in the one-dimensional space of the viral DNA, greatly reduces the number of possible orientations of AVP and its precursor protein substrates relative to each other, compared to both being free in solution. It is possible that the orientation of AVP-pVIc complexes sliding on DNA and the orientation of their substrates also bound to DNA are such that almost every collision between enzyme and substrate will be productive, *i.e.*, lead to catalysis. This one-dimensional biochemistry, in a crowded milieu where DNA defines a highway through space, may be the only way bimolecular reactions between proteins can occur efficiently inside a virus particle or even in the nucleus of a cell.

### 6.2. Role of Maturation in the AdV Infectious Cycle

*Goals for virion maturation.* There is a double goal for maturation on the viral cycle: first, to produce virions stable enough to protect the genome from aggressive conditions in the extracellular milieu; and second, to prepare the viral particle for correct delivery of the genome into the new host cell. In AdV, maturation prepares the particle for a programmed uncoating sequence upon reception of the appropriate signal, for example attachment to the receptor, or pH changes along the endocytosis pathway [128,131,134]. Interestingly, AdV maturation is related to genome packaging in a unique way, determined by the use of dsDNA as a fundamental cofactor in the function of AVP. Additionally, a protein required for genome packaging, L1 52/55k, is also a substrate for the protease and its processing is the mechanism used to remove it from the particle [60]. Scaffold release triggered by proteolysis is a common mechanism encountered in other dsDNA viruses [125]. Adenovirus may use a dual scaffolding system, including both a separate protein and flexible regions of minor capsid proteins removed by the viral protease during maturation.

*Temporal sequence of events.* One of the many remaining questions regarding AdV assembly, packaging and maturation is the temporal sequence of events. While only negligible amounts of L1 52/55k are found in mature particles, *in vitro* proteolytic processing yields large fragments of this protein (17 kDa minimum), suggesting that large openings must exist in the nascent virion for them to be released [60]. On the other hand, excised peptides of other AVP substrates remain trapped in mature virions [19,53,129]. From these observations, it could be hypothesized that maturation would occur in two different phases: one simultaneously with packaging, through some openings which may or may not be also used for genome translocation; and another after packaging and L1 52/55k release, when the viral particle is already sealed.

*Roles of precursor proteins and their cleavage products in maturation.* Structural and biophysical analyses indicate that cleavage of all AVP substrates results in a metastable particle [128,131,134]. However, the exact role of each particular cleavage or of each particular precursor in determining particle stability and infectivity is not known. Mutation studies where each cleavage is separately impaired are required to elucidate this point. To understand these roles in detail, it is essential to progress in the structural studies. First, to overcome the current uncertainties regarding the location of the different minor coat proteins in the icosahedral shell [8,10]; second, to start obtaining data on the organization of non-icosahedral capsid components. This last point is particularly relevant to the maturation process, as many of the AVP substrates (Figure 1) are not icosahedrally ordered. One particularly intriguing example is polypeptide pVI. This protein must slide on the viral genome until it finds AVP to activate the protease and trigger the maturation cleavage cascade [108]. However, pVI is bound to hexon in the icosahedral shell [10,104,127,128], therefore being unable to slide. Is it possible that there are two different pVI pools during assembly, one interacting with hexons, the rest free to slide on DNA and activate AVP? The disparity between the copy number of VI in the virion (360) and that of hexon (720 monomers, 240 trimers) may be hinting at such protein distribution. Latest advances in the field of cryo-electron microscopy [144] or mass spectrometry of large complexes [145] will likely be crucial in addressing these challenging questions.
